# Supplementary material for: Pharmacological inhibition of tyrosine protein-kinase 2 reduces islet inflammation and delays type 1 diabetes onset in mice
Source: bioRxiv. 2024 May 9:2024.03.20.585925. Preprint. [Version 2] doi: 10.1101/2024.03.20.585925 (PMC11100605; doi:10.1101/2024.03.20.585925)

Supplemental Figure 1

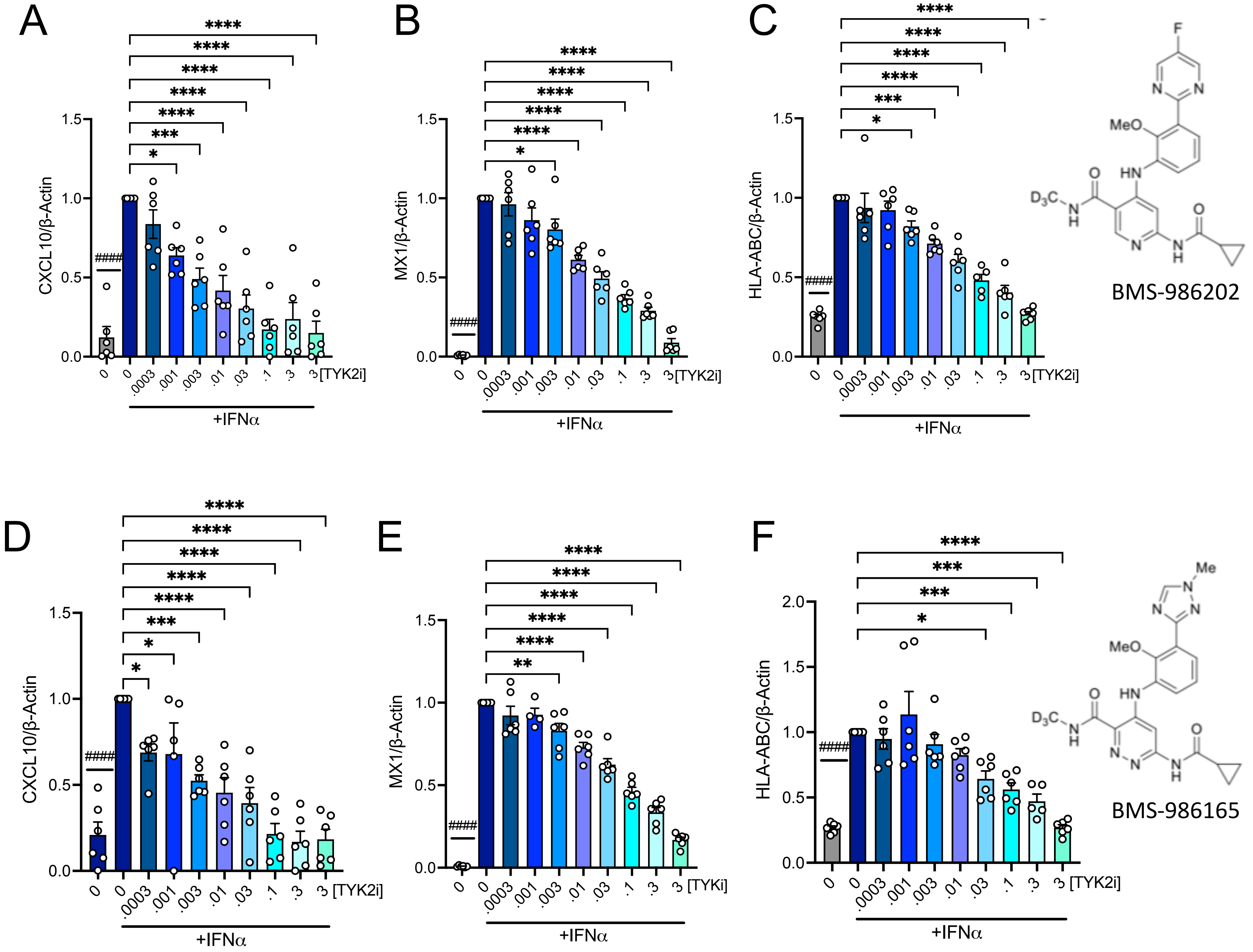

Supplemental Figure 2

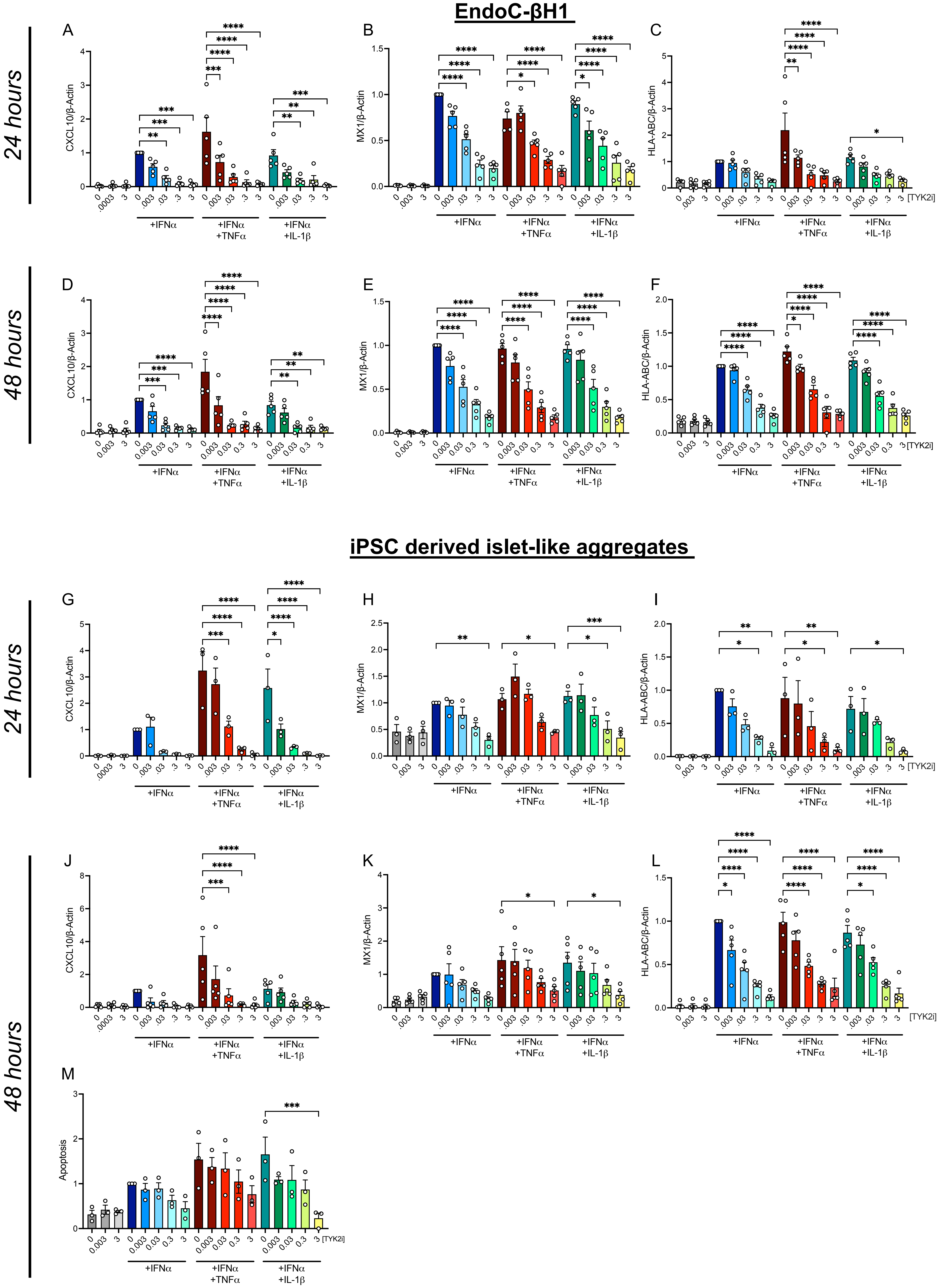

Supplemental Figure 3

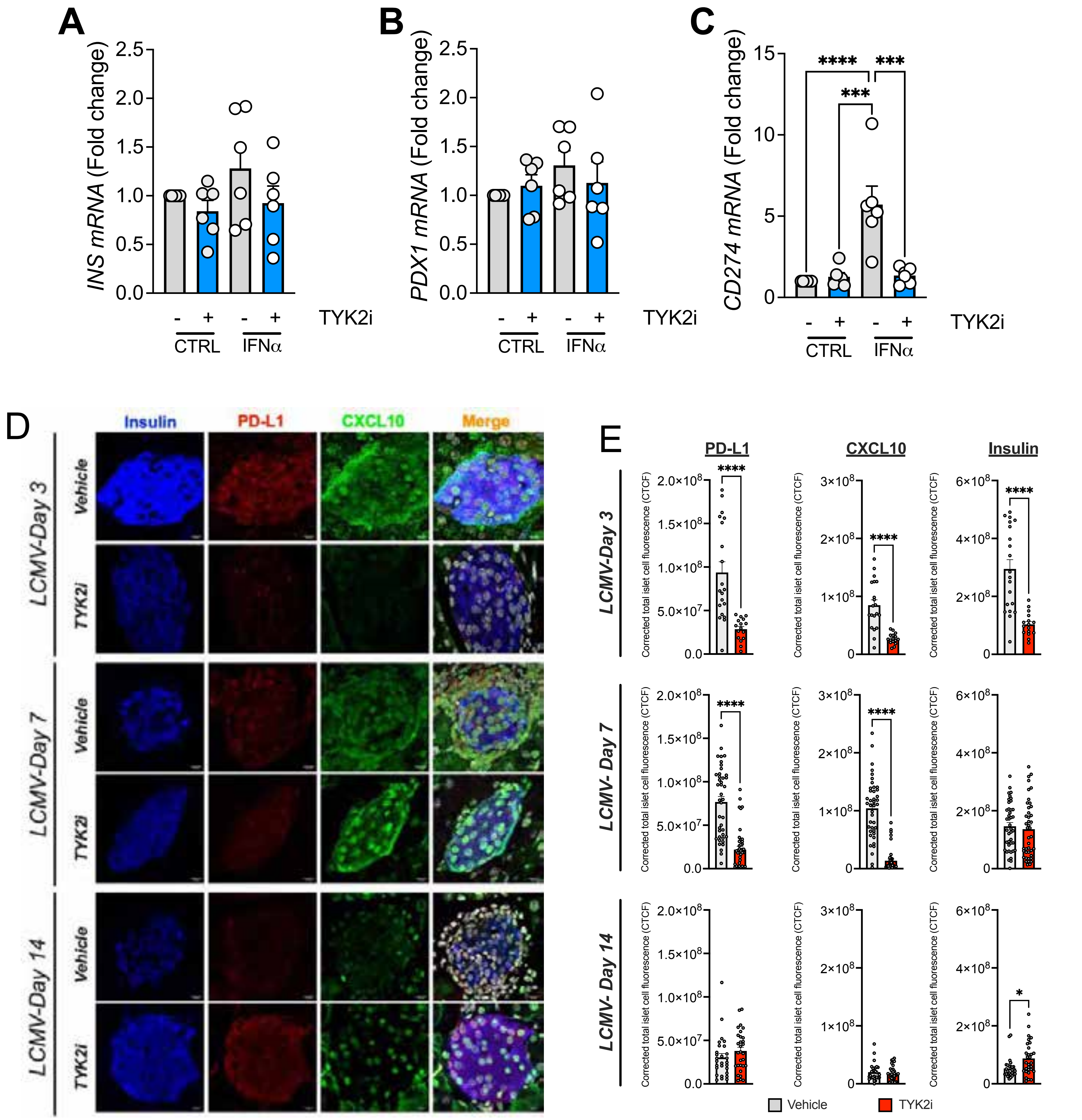

Supplemental Figure 4

Innate panel

A

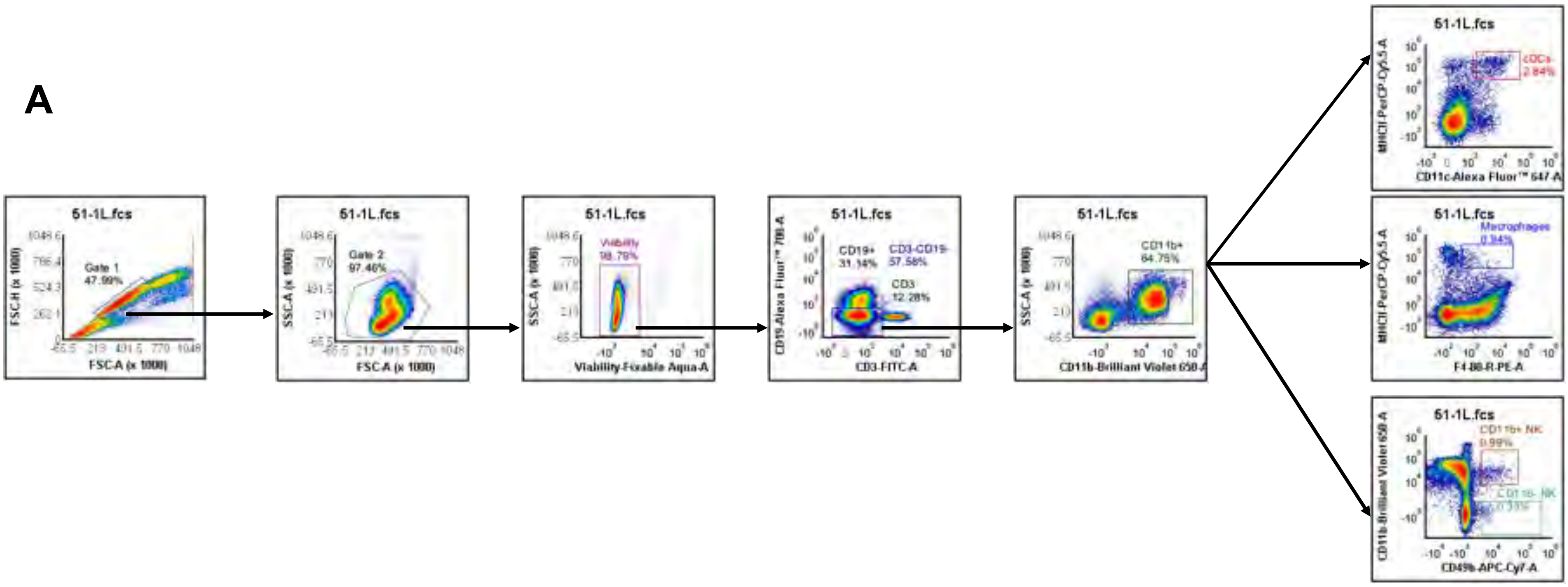

Adaptive panel

B

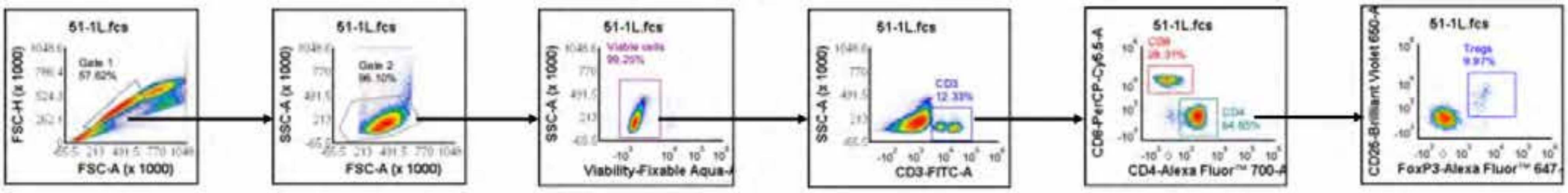

Supplemental Figure 5

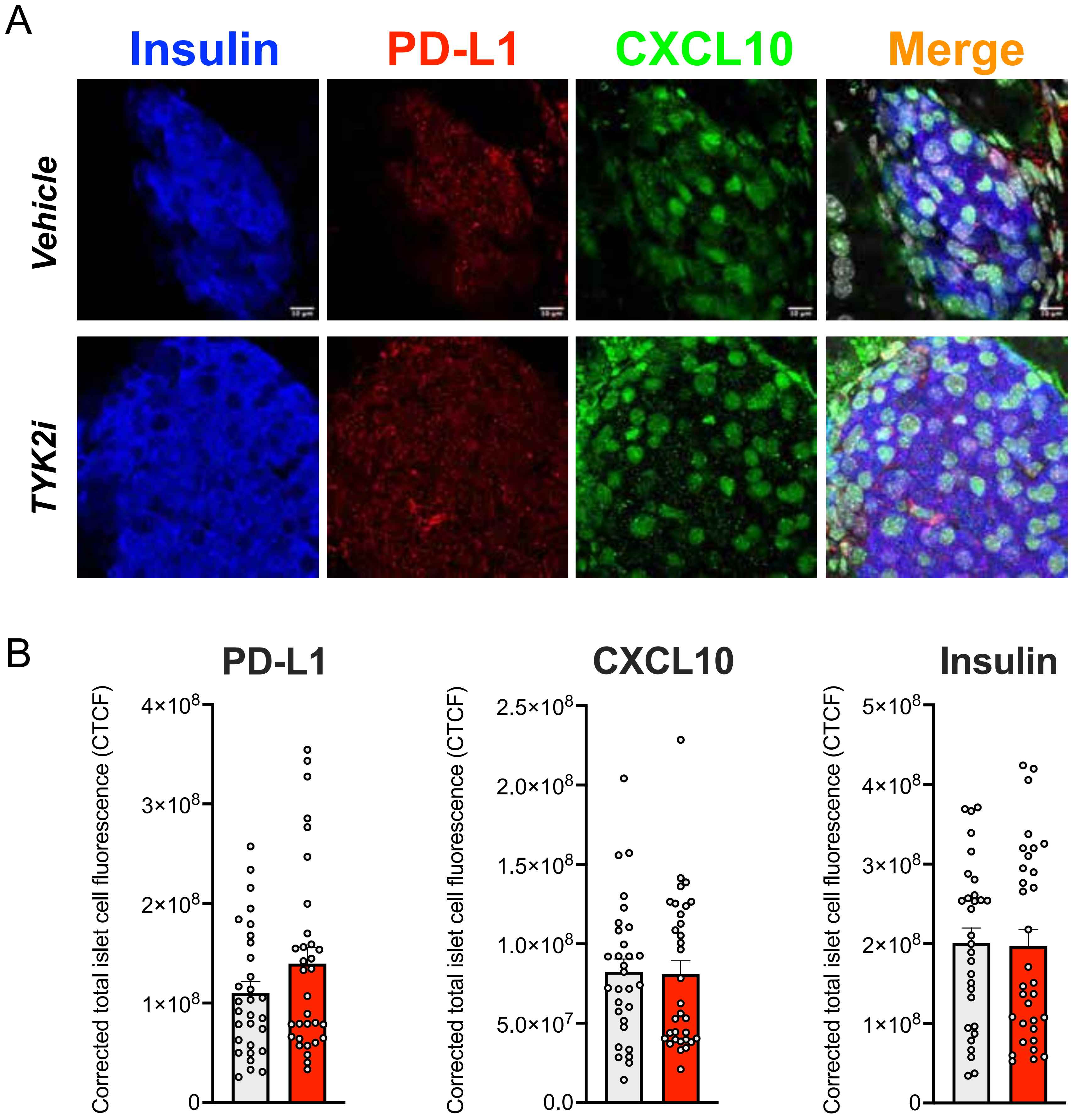

Supplemental Figure 6

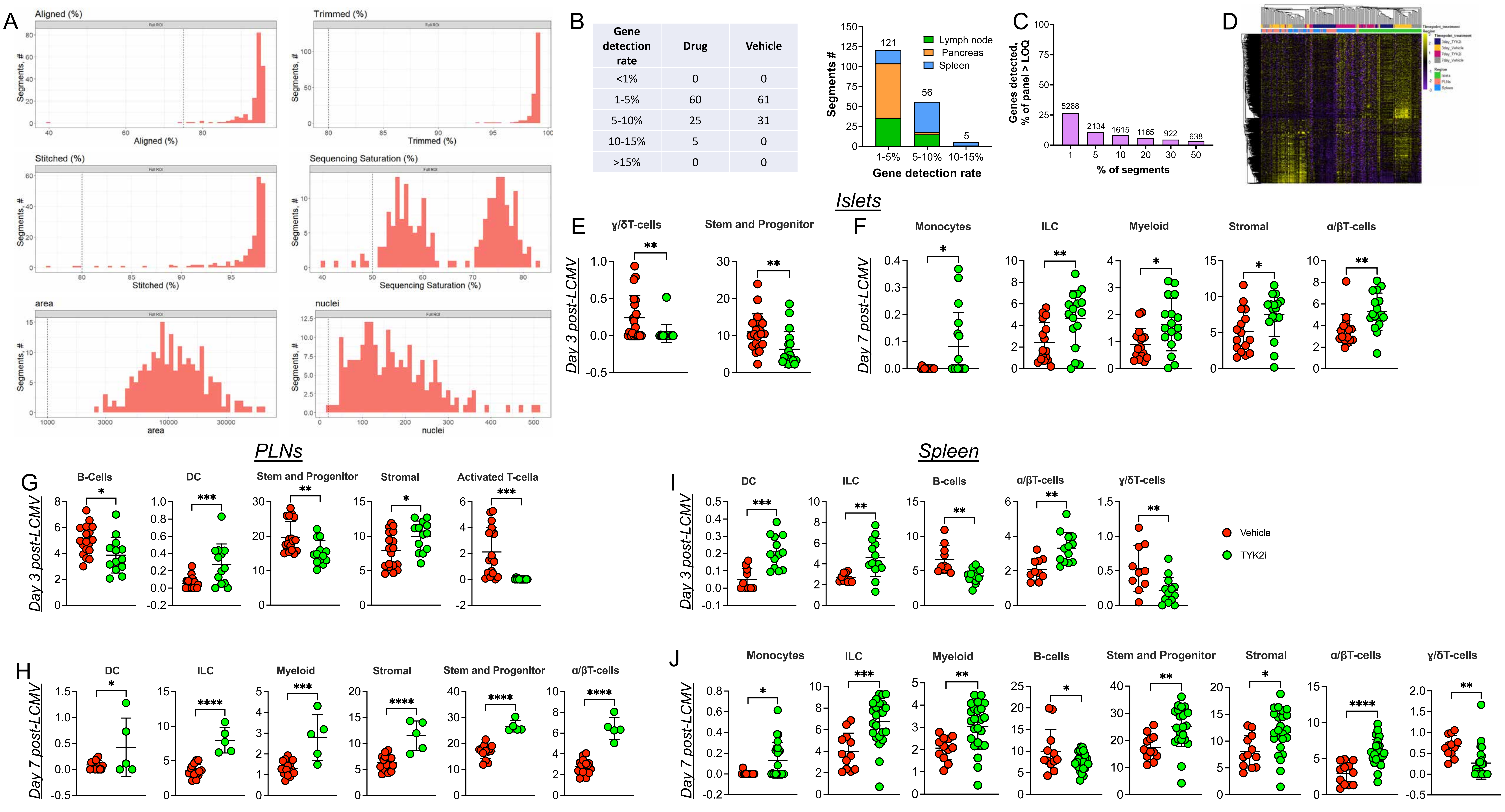

Gene detection rate	Drug	Vehicle
<1%	0	0
1-5%	60	61
5-10%	25	31
10-15%	5	0
>15%	0	0

Supplemental Figure 7

A

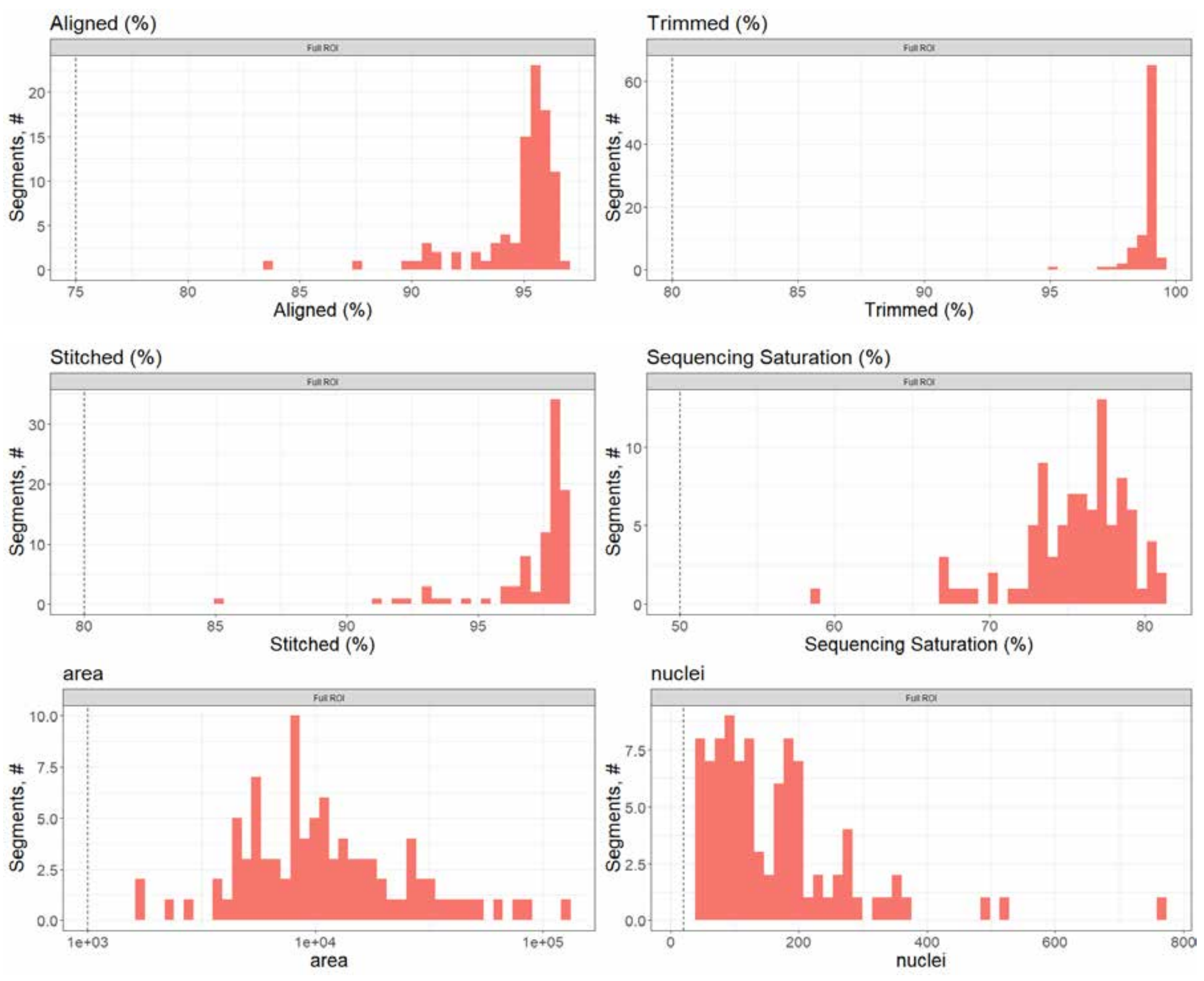

B

Gene detection rate	Drug	Vehicle
<1%	0	0
1-5%	0	18
5-10%	1	19
10-15%	0	8
>15%	45	1

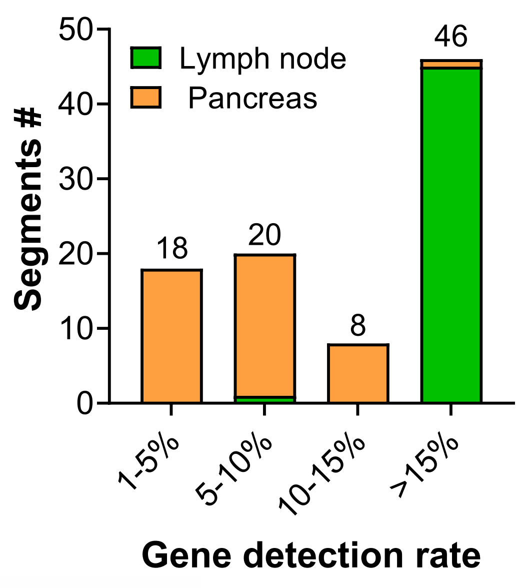

C

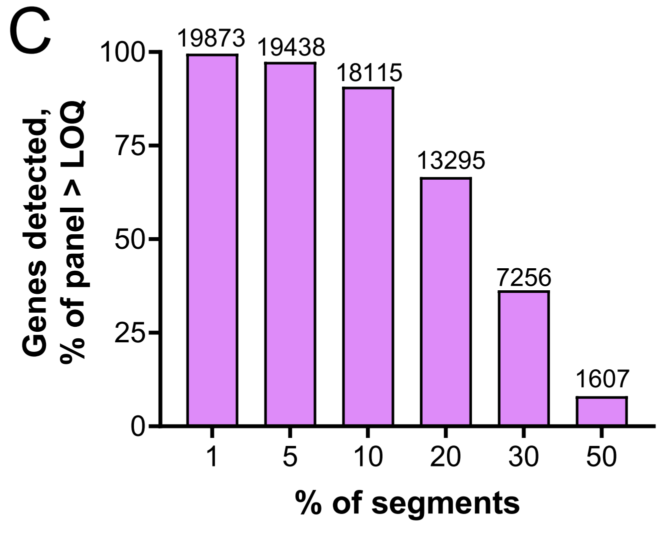

D

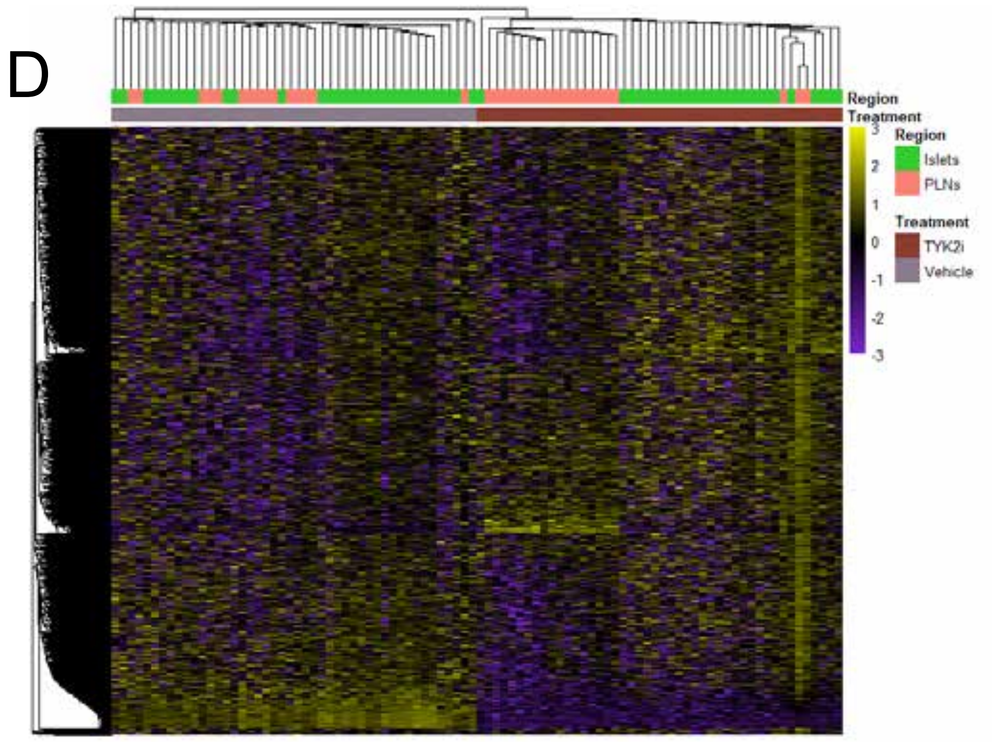

Supplemental Figure 8

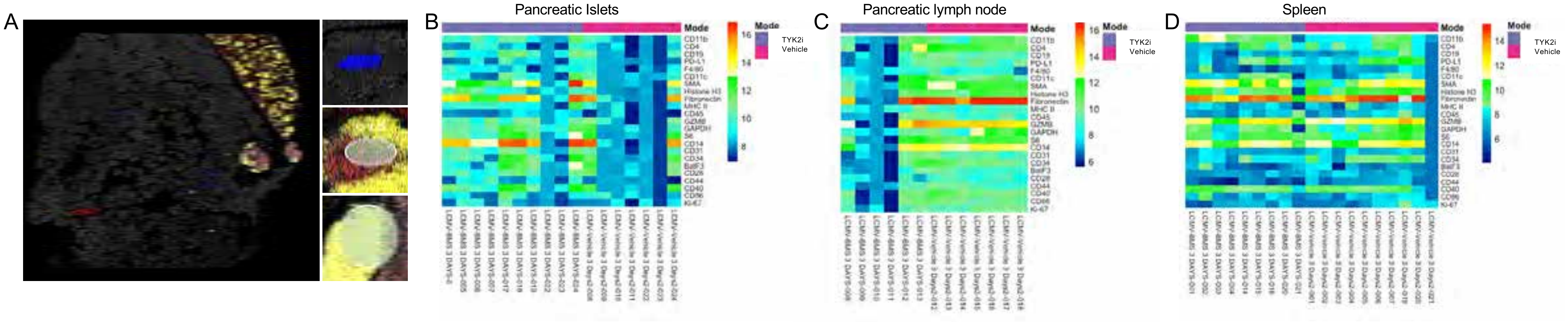

Supplement: 1 [file NIHPP2024.03.20.585925V2-supplement-1.pdf]
